# A prospective investigation of predictive and modifiable risk factors for breast cancer in unaffected *BRCA1* and *BRCA2* gene carriers

**DOI:** 10.1186/1471-2407-13-138

**Published:** 2013-03-21

**Authors:** Emer M Guinan, Juliette Hussey, Sarah A McGarrigle, Laura A Healy, Jacintha N O’Sullivan, Kathleen Bennett, Elizabeth M Connolly

**Affiliations:** 1 Discipline of Physiotherapy, School of Medicine, Trinity Centre for Health Sciences, St. James’s Hospital, Dublin, Ireland; 2 Department of Surgery, Trinity Centre for Health Sciences, St. James’s Hospital, Dublin, Ireland; 3 Department of Clinical Nutrition, St. James’s Hospital and Trinity College Dublin, Dublin, Ireland; 4 Department of Pharmacology and Therapeutics, Trinity Centre for Health Sciences, St. James’s Hospital, Dublin, Ireland; 5 Department of Surgery, St. James’s Hospital, Dublin, Ireland

**Keywords:** *BRCA1*, *BRCA2*, Breast cancer, Metabolic syndrome, Physical activity, Body composition, Dietary intake, Telomere length

## Abstract

**Background:**

Breast cancer is the most common female cancer worldwide. The lifetime risk of a woman being diagnosed with breast cancer is approximately 12.5%. For women who carry the deleterious mutation in either of the *BRCA* genes, *BRCA1* or *BRCA2*, the risk of developing breast or ovarian cancer is significantly increased. In recent years there has been increased penetrance of *BRCA1* and *BRCA2* associated breast cancer, prompting investigation into the role of modifiable risk factors in this group. Previous investigations into this topic have relied on participants recalling lifetime weight changes and subjective methods of recording physical activity. The influence of obesity-related biomarkers, which may explain the link between obesity, physical activity and breast cancer risk, has not been investigated prospectively in this group. This paper describes the design of a prospective cohort study investigating the role of predictive and modifiable risk factors for breast cancer in unaffected *BRCA1* and *BRCA2* gene mutation carriers.

**Methods/design:**

Participants will be recruited from breast cancer family risk clinics and genetics clinics. Lifestyle risk factors that will be investigated will include body composition, metabolic syndrome and its components, physical activity and dietary intake. PBMC telomere length will be measured as a potential predictor of breast cancer occurrence. Measurements will be completed on entry to the study and repeated at two years and five years. Participants will also be followed annually by questionnaire to track changes in risk factor status and to record cancer occurrence. Data will be analysed using multiple regression models. The study has an accrual target of 352 participants.

**Discussion:**

The results from this study will provide valuable information regarding the role of modifiable lifestyle risk factors for breast cancer in women with a deleterious mutation in the *BRCA* gene. Additionally, the study will attempt to identify potential blood biomarkers which may be predictive of breast cancer occurrence.

## Background

Breast cancer is the most common female malignancy worldwide. The lifetime risk of a woman being diagnosed with breast cancer is approximately 12.5% [1]. While most breast cancers are sporadic in nature, approximately 5-10% are attributed to genetics, arising from autosomal dominant mutations in specific cancer genes, the strongest of which are the two breast cancer susceptibility genes, *BRCA1* or *BRCA2*. Women who carry these mutations have up to an 80% risk of developing breast and up to a 60% risk of ovarian cancer [2-4]. In recent years, there has been increased penetrance of *BRCA1*/2 mutations, which is most likely mediated by lifestyle or environmental influences [2,5,6], prompting investigation into the potential of reducing risk through lifestyle modification in this group. Understanding how modifiable and lifestyle risk factors affect cancer risk, specifically in *BRCA* mutation carriers, may have important implications for cancer prevention in this high risk group.

The associations between obesity, physical inactivity and certain components of dietary intake such as alcohol consumption and sporadic breast cancer risk are well established [7-10]. Obesity may increase breast cancer risk through a number of different mechanisms including insulin resistance, the metabolic syndrome, increased production of sex hormones, insulin-like growth factors, chronic low-grade inflammation and alterations in adipokines[9,11-15]. These biological pathways, in turn, are the hypothesised targets through which physical activity may exert its protective effects over breast cancer development [16,17]. However, knowledge regarding the role of these lifestyle factors in *BRCA1/2* mutation carriers is limited.

A number of case-control studies have investigated the association between adult weight change and breast cancer risk in *BRCA* gene mutation carriers through self-reported recall of lifetime weight changes [2,18-20]. Healthy weight during adult life, particularly from menarche to 21 years, has been associated with decreased breast cancer risk [18,20] while weight loss between 18 and 30 years of age has been associated 34% reduction in breast cancer risk, particular among *BRCA1* mutation carriers [18]. Consistent with sporadic breast cancer, menopausal status is a potentially modifying factor in the relationship between obesity and breast cancer risk in *BRCA* mutation carriers, however results are conflicting. A study by Kotsopoulos and colleagues [18], reported increased breast cancer risk with adult weight gain, regardless of menopausal status, while Manders *et al.,*[19] reported an association with postmenopausal breast cancer only. However, in these studies, body weight has been self-reported, rather than objectively measured, leading to potential inaccuracies in results. The association between physical activity and *BRCA* mutation-associated breast cancer is unclear with two studies reporting a protective effect [2,21] and one showing no association [20]. However, as with the anthropometric variables, measurement methods were limited and relied on self-reported recall.

In addition, it has been suggested that measuring telomere length may help predict risk of occurrence of certain types of cancer [22-24] including *BRCA* mutation-associated breast cancer [25,26]. There is some evidence to suggest that exercise may affect telomere length. For example, Puterman *et al,* showed that increased perceived stress was associated with increased odds of having short telomeres but only in non-exercising women [27]. Non-exercising women with a history of childhood abuse had shorter telomeres than those with no history of abuse; however, in women who exercised regularly no link between childhood abuse and telomere length was found [28]. Together, these findings suggest that telomere shortening may be modifiable by physical activity.

Reproductive factors including parity and breastfeeding practices have been associated with risk reduction in *BRCA* mutation carriers similar to that of the general population [29,30], suggesting that modifiable risk factors can attenuate risk in this group. However, our understanding of the potential for risk reduction for the majority of modifiable of risk factors in this high risk group remains unknown. Studies investigating women with a strong family history of breast cancer have shown that these women are no more likely to engage in healthy lifestyle habits than women in the general population [31,32] making lifestyle interventions a potentially important target. The Consortium of Investigators of Modifiers of *BRCA1/2* (CIMBA) is currently examining the role of various genetic modifiers of cancer risk in *BRCA* mutation carriers. Within CIMBA, and as part of the Epidemiological Study of Familial Breast Cancer (EMBRACE) study and others, the influence of lifestyle factors on breast cancer occurrence will be measured subjectively using a lifestyle questionnaire (http://ccge.medschl.cam.ac.uk/embrace/). However these associations have not been investigated using validated and objective measures of lifestyle parameters.

We plan to prospectively and objectively examine the association between modifiable (body composition, metabolic syndrome, physical activity and dietary intake) and potentially predictive (telomere length) risk factors for breast cancer and breast cancer occurrence in unaffected *BRCA1* and *BRCA2* gene mutation carriers. This study will also investigate the hypothesised link between physical activity and telomere length in a cohort of unaffected *BRCA*-mutation carriers. Various correlations between telomere length and physical activity, lifestyle factors and breast cancer occurrence will be examined.

## Methods / design

### Study design

This study is designed as a prospective cohort study aiming to evaluate the role of modifiable and predictive risk factors for breast cancer in women who have been genetically tested and identified as carriers of a deleterious mutation in either one of the *BRCA* genes, *BRCA1* or *BRCA2*.

### Objectives

1. To determine information on metabolic syndrome, body composition, physical activity, diet, telomere length and hormone measurements from women who are unaffected *BRCA1/2* mutation carriers.

2. To assess the associations between the following with body composition, physical activity and dietary intake in unaffected *BRCA1/2* mutation carriers:

• Insulin resistance

• Leptin

• Adiponectin

• Inflammatory markers

• Telomere length

3. To prospectively examine the relationship between the metabolic syndrome, body composition, physical activity, diet, telomere length, and hormone measurements with breast cancer occurrence among *BRCA1/2* mutation carriers.

### Participant recruitment

A convenience sample of *BRCA* gene mutation carriers will be recruited from St. James’s Hospital, Dublin and the National Centre for Medical Genetics, Our Lady’s Children’s Hospital, Crumlin, Dublin. Potentially suitable participants, who have previously undergone genetic testing and have been identified as carriers of either the *BRCA1* or *BRCA2* mutation, will be identified at Breast Cancer Family Risk clinics and Cancer Genetics clinics, by medical teams and specialised breast cancer and medical nurses. At the St. James’s Hospital site, potentially suitable participants will be provided with information leaflets about the study during routine medical consultations, and if interested in gaining further information, will be directed to speak to a member of the research team who will attend the clinic. At the National Centre for Medical Genetics, Our Lady’s Children’s Hospital, Crumlin potentially suitable participants will be provided with information leaflets detailing the study during medical appointments and invited to contact the research team by email or by telephone if interested in gaining further information. Study personnel will provide further information to interested participants and assess for eligibility. Ethical approval has been granted by the SJH/AMNCH Joint Hospital Research Ethics Committee, in accordance with the Helsinki declaration, and written informed consent will be obtained from all participants.

Participants who meet the following criteria will be eligible to participate:

i. Women who have been genetically tested and identified as carriers of a deleterious mutation in either one of a *BRCA* genes, *BRCA1/2 *and who do not have a history or either breast or ovarian cancer.

ii. Aged 18 years or above

iii. Able to understand English.

iv. Willing to travel to the study site for measurements.

Participants will be excluded for the following reasons:

i. History of cancer or evidence of active disease (exception: non-melanoma skin cancer).

ii. Confirmed pregnancy or history of childbirth in the preceding 6 months. Women who become pregnant during the study period will complete follow-up active assessments at least 6 months after giving birth.

iii. Any medical co-morbidity that would preclude the ability to participate in the study

iv. Dependence on a mobility assistive device.

v. Participants, who at the local investigator’s discretion are not thought appropriate e.g. very upset and emotional regarding finding of *BRCA* gene mutation, family, work or transport issues that would make participation difficult.

### Assessments

**Figure 1:**
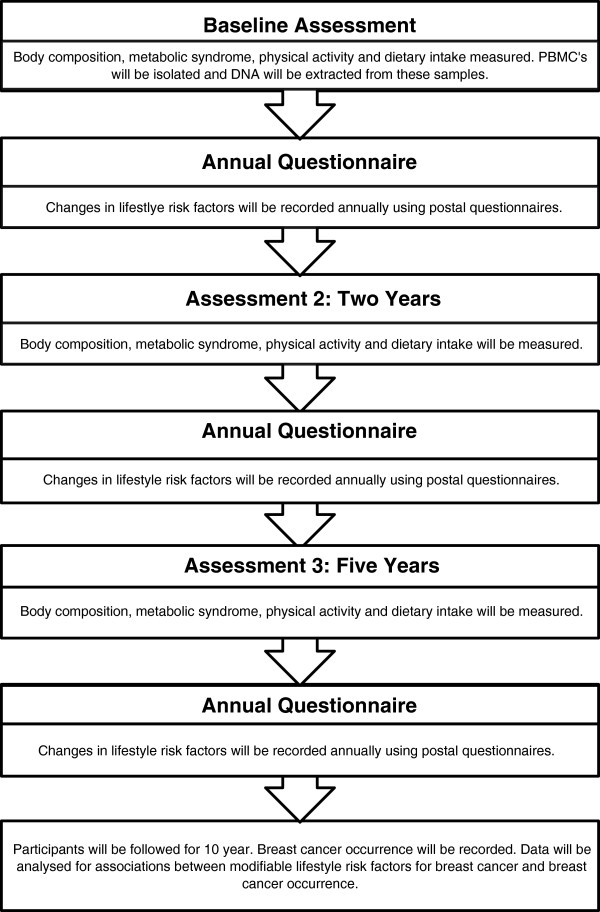
**Flow of data collection throughout the study.** Active assessments will be completed at baseline, two years and five years. Annual questionnaires will be sent by post to participants following enrolment to monitor change in lifestyle risk factors being measured.

Participants will complete three active assessments during the study (baseline/entry to the study, two-years, and five-years) during which all measurements outlined below will be completed. Information on disease occurrence, risk reducing procedures and current lifestyle habits will be gathered yearly for 10 years using posted questionnaires. The flow of data collection throughout the study is shown in Figure 1.

### Baseline characteristics

Demographic details will be gathered from medical charts and through patient interview. Details on past medical history, breast cancer risk factors (family history of breast cancer, oral contraceptive use, age at first birth, menopausal status, parity), and socio-demographic variables including smoking history, alcohol habits, employment status and marital status will be collected.

### Body composition

Anthropometric data will be collected following a 12 hour fast. Standing height will be measured, without shoes, to the nearest millimetre using SECA stadiometer. Body composition will be estimated using a bioimpedance analyser, the Tanita MC 180 MA Multi-Frequency Body Composition Analyzer (Tanita Corp, Tokyo, Japan). Data output from the Tanita will be recorded and will include body weight, body mass index (BMI), percentage body fat, muscle mass and fat free mass. Waist circumference will be measured using a flexible measuring tape, in duplicate, to the nearest millimetre, at the mid-point between the top of the iliac crest and the last rib [33]. Lifetime weight changes will be assessed at the initial assessment by asking participants to recall their birth weight, weight at menarche, weight at age 18, 21, 30 and 40 respectively.

### Blood pressure

Resting blood pressure will be measured using the auscultatory method in accordance with the Joint National Committee on Prevention, Detection, Evaluation, and Treatment of High Blood Pressure guidelines [34]. Measurements will be taken in duplicate and the mean taken for data entry.

### Venous sampling

#### Metabolic profile

Venous blood samples will be collected in the morning following a 12-hour fast. Participants will be asked to refrain from moderate-vigorous intensity exercise for 24 hours prior to collection. Samples will be taken to measure glucose, insulin, lipid profile (total cholesterol (TC), high-density lipoprotein-cholesterol (HDL-C), low-density lipoprotein cholesterol (LDL-C) and triglycerides (TG)), glycosylated haemoglobin levels (HBA1c), C - reactive protein (CRP), leptin, total adiponectin and sex hormone binding globulin. Insulin resistance will be estimated using the Homeostatic Model Assessment: [(Fasting glucose (mmol.L^-1^) x fasting insulin (mU.L^-1^))/22.5] [35].

Many biomarkers of carcinogenesis must be collected and stored prior to cancer occurrences, to ensure an unequivocal link between biomarker exposure and tumorgenesis [36]. Previous studies examining lifestyle factors in this group have adopted a case-control design and therefore have not facilitated this type of analysis. In the current prospective study, serum and plasma samples will be collected and stored in a biological bank at -80°C for future analysis of associations between carcinogenesis and biological markers of obesity, physical activity and diet.

### Telomere length

Peripheral blood mononuclear cells (PBMCs) will be isolated from venous blood by density centrifugation using Ficoll-Paque™ Plus (GE Healthcare, Uppsala, Sweden). Genomic DNA will be extracted from PBMCs by standard procedures. Telomere length will be measured in extracted genomic DNA by quantative polymerase chain reaction (qPCR) using a method adapted from the one origanally described by Cawthon [37]. Briefly, two PCRs will be performed for each sample: one to amplify the telomeric DNA and a second to amplify a single-copy control gene (36B4, acidic ribosomal phosphoprotein PO). This provides an internal control to normalize the starting amount of DNA. A five-point standard curve (2-fold serial dilutions from 10 to 0.625 ng of DNA) will be included on all plates to allow the transformation of Ct (cycle threshold) into nanograms of DNA. All samples will be run in triplicate and the median will be used for subsequent calculations. A relative measurement of the telomere length of each sample will be calculated by dividing the amount of telomeric DNA by the amount of control-gene DNA. Two control samples will be run in each experiment to allow for normalization between experiments and periodical reproducibility experiments will be performed to guarantee correct measurements.

Genomic DNA samples (50 ng) will be amplified in a total reaction volume of 20 μl containing 2X Quantifast ™ SYBR green PCR master mix (Qiagen Inc., CA, USA), 1 μl of forward and reverse primer (Metabion, Germany) and 7 μl of DNase free water. For the telomere amplification PCR, 300 nM of each primer (tel1b: CGGTTTGTTTGGGTTTGGGTTTGGGTTTGGGTTTGGGTT; tel2b: GGCTTGCCTTACCCTTACCCTTACCCTTACCCTTACCCT) will be used. The thermal cycling profile for the telomere amplification will be 30 cycles of amplification at 95°C for 15 s and at 56°C for 60 s. For the control gene amplification, 300nM of forward primer (36B4u: CAGCAAGTGGGAAGGTGTAATCC) and 500nM (36B4d: CCCATTCTATCATCAACGGGTACAA) of reverse primer will be used. The thermal cycling profile in this instance will be 35 cycles of amplification at 95°C for 15 s and at 56°C for 20 s and 72°C for 20 s. Both PCR reactions will require an initial denaturation step at 95°C for 15 min. Threshold cycle (Ct) values for each sample will be converted into nanograms of DNA using standard curves. Ct values from the telomere assay will be normalized to the single gene reference. The telomere length (x) from each sample will be calculated as the telomere to single copy gene ratio (T/S ratio) and will be based on the calculation of the ΔC_T_ [C_T_(telomere)/C_T_(single gene)]. Telomere length (expressed as a relative T/S ratio) will be normalized to the average T/S ratio of the reference sample.

### Metabolic syndrome classification

The metabolic syndrome will be diagnosed in the presence of any three of the following: elevated waist circumference (≥80 cm); elevated TG (≥1.7 mmol.L^-1^) or drug therapy for lipid abnormalities; reduced HDL-C (<1.3 mmol.L^-1^) or drug therapy for lipid abnormalities; elevated blood pressure (systolic ≥130 mmHg and/or diastolic ≥85 mmHg) or antihypertensive medication; elevated fasting glucose (≥100 mg.dL^-1^) or glucose-lowering medication [38].

### Physical activity

Current physical activity levels will be objectively measured using the RT3 activity monitor (Stayhealthy Inc. Montrovia, California, USA). The RT3 activity monitor is a small, lightweight, battery operated device, designed to measure accelerations along three orthogonal planes (triaxial accelerometer). Participants will wear the monitor for seven days, during waking hours, following each active assessment (baseline, two-years and five-years). Participants will be provided with the monitor following completion of each assessment and will be given detailed written and verbal instruction on its use. Participants will also be provided with a stamped addressed envelope to return the monitor to the centre after one week (battery life of the monitor = 21 days). The validity [39] and reliability [40] of the accelerometer have been established. The output from the RT3 activity monitor will provide objective quantification of sedentary, light, moderate and vigorous intensity activity, while also facilitating analysis for adherence to activity guidelines.

Physical activity during the preceding 12 months will be estimated using two quantitative history questionnaires, The Minnesota Leisure-Time Physical Activity Questionnaire (MLTPAQ) and the Tecumseh Occupational Physical Activity Questionnaire (TOPAQ). The MLTPAQ is an interview administered questionnaire, measuring leisure-time physical activities [41] while the TOPAQ can be either self-administered or interview based and focuses on occupation-related activities of a maximum of three jobs [42]. Both questionnaires measure frequency of activity during the preceding year in terms of months and time per occasion, and combine this information with intensity scores to measure physical activity in terms of metabolic-equivalent minutes per day (MET-min/day)[41,43]. The MET-score can be derived from the Compendium of Physical Activities [44], and values can be updated accordingly during the study period. The questionnaires will be interview-administered at each of the three active assessment points (baseline, two-years and five-years). The validity of both questionnaires have been well established in a range of populations [41,42,45,46], with one study reporting significant correlations between cardiorespiratory fitness and both leisure-time activity and household activity in 375 middle-aged women [47]. Both the MLTPAQ and the TOPAQ will be completed during each of the three active assessments, through interview, according to standardised interview guidelines [41].

Finally, the Godin Leisure-Time Exercise Questionnaire will be incorporated into the annual questionnaire. Participants will be required to complete this questionnaire independently, but will be informed to contact study personnel should issues arise. The Godin is a simple, self-administered, four-item questionnaire that is designed to measure an individual’s leisure-time activity during a typical week to provide a global impression of an individual’s activity status [48]. The validity and reliability of the questionnaire have been established [46,48] and the questionnaire has been widely used in cancer research [49,50].

### Dietary intake

Dietary intake will be assessed using two different methods, in this prospective cohort study on modifiable risk factors for *BRCA1* and *BRCA2* associated breast cancer, “current” intake will be assessed using food diaries and “habitual” or usual intake assessed by a food frequency questionnaire (FFQ).

Participants will be asked to complete an open-ended estimated 3 day food diary following each of the three active assessments. Participants will be provided with the diary at the end of each assessment and receive detailed verbal and written instructions detailing how it should be completed. The diet-diary booklet contains clear instructions of how to complete the food diary, as well as a detailed good and poor example of food intake. The instructions indicate that the respondent should record the food brand, portion size, cooking methods and includes a prompt for the name and daily dose of any vitamin, mineral or food supplements taken each day. General questions, for example, on the type of milk, spreadable fat usually consumed, and salt use will be asked as part of a general food habits questionnaire. Participants will be given a prepaid envelope to return the completed diary. The energy intake: calculated BMR ratio [51] will be used as a measure of the degree of energy underreporting with each dietary method.

The FFQ used in this prospective study is a self-administered Willett FFQ adapted from the European Prospective Investigation of Cancer (EPIC) study [36] and was used in the Irish Survey of Lifestyle, Attitudes and Nutrition (SLÁN) 2007 [52]. This FFQ has previously been validated for use in an Irish adult population [53]. The FFQ consists of a checklist of 149 food and beverage items divided into the following main food groups consumed in Ireland; bread and savoury biscuits; cereals; potatoes, rice and pasta; dairy products and fats, meat fish and poultry; fruit; vegetables; sweets and snacks; soups, sauces and spreads and lastly drinks. Participants will be asked to report how often each food item was consumed during the previous year using common units or portion size for each food, e.g. one egg or one slice of bread will be specified. The nine frequency responses range from ‘never or less than once per month’ to ‘six or more times per day’. Calculations for nutrient intake can be estimated via computerized software programs that multiply the reported frequency of each food by the amount of nutrient in a serving of that food. The FFQ will be incorporated into the annual questionnaire and completed by participants independently at home. Participants will be encouraged to contact study personnel if issues arise.

### Annual questionnaire

Information on cancer occurrence and potential confounding factors affecting breast cancer risk will be gathered using questionnaires which will be completed on an annual basis after participants have enrolled. The questionnaire aims to track changes in modifiable lifestyle risk factors (weight, smoking, alcohol use, physical activity and diet), risk reducing procedures (prophylactic surgery), reproductive factors (childbirth, breastfeeding, use of oral contraceptive pill or hormonal replacement therapy) or cancer occurrence.

### Statistical analysis

*Sample size calculation*: There is some evidence to suggest an association between BMI and breast cancer incidence in *BRCA* mutation carriers [19-21] and even stronger evidence to suggest an association with sporadic breast cancer [7]. Therefore BMI was the chosen outcome on which the power analysis is based. Assuming a difference of in BMI of 15 kg.m^-1^ between cases and controls, with a relative risk of 2.08, 80% power and two-sided 5% significance level 141 participants would be required per group. However, a minimum sample size of 352 was calculated to allow for a 25% drop out rate due to the long-term nature of this study.

Data will be analysed using the SPSS package for Windows. Baseline descriptive statistics will be presented as means (standard deviations) for normally distributed continuous data, medians (inter quartile range, IQR) for non-normal data and as frequency (percentage) for categorical variables. Distributions will be checked for normality using the K-S test and non-normal data will be transformed using appropriate transformations. Differences in means of continuous variables (waist circumference, blood pressure (systolic and diastolic), lipids (TC, HDL-C, LDL-C and TG), glucose, insulin, HBA1c, CRP, leptin, adiponectin, BMI, fat free mass, percentage body fat, muscle mass, percentage time in each domain of physical activity, energy intake, telomere length) will be compared across categories (BMI sub-groups, presence or absence of metabolic syndrome, adherence to physical activity guidelines) using independent sample t-tests or ANOVA as appropriate for normally distributed variables and the Mann Whitney U test (or Kruskal-Wallis test) for non-normally distributed data. Chi-squared analysis will be used to compare categorical variables across the above stated groups.

Pearson or Spearmans correlation analysis will be conducted between body composition (BMI, waist circumference, percentage body fat), physical activity and dietary intake and the following variables: insulin resistance, leptin, adiponectin, inflammatory markers and telomere length for normally and non-normally distributed data respectively. Variables found to be associated at p<0.10 will be examined further using separate multiple regression analyses with body composition, physical activity and dietary intake as the dependent variables.

Cox proportional hazards regression models will be used to compute adjusted hazard ratios of time to breast cancer (event) with 95% confidence intervals. Data will be censored at the last available follow-up where the breast cancer status was recorded. Continuous variables will be categorised into known cut-points based on previous research (BMI will be categorised as <18.5 kg.m^-2^, 18.5-24.9 kg.m^-2^, 25-29.5 kg.m^-2^, 30-34.9 kg.m^-2^, 35-39.9 kg.m^-2^, >40 kg.m^-2^; waist circumference will be categorised as <80 cm, 80-87.9 cm, >88 cm; physical activity will be categorised as adherence to physical activity guidelines (30 minutes moderate intensity activity, 5 days per week) and non-adherence to activity guidelines). Quartile cut-offs will be established for markers without pre-determined categories. Metabolic syndrome variables (waist circumference, blood pressure, HDL-C, TG and glucose) will be standardized to z-score variables with mean=0, SD=1 and a composite z-score will be computed for the presence/absence of the metabolic syndrome. Confounding variables that will be considered in the analysis include: prophylactic surgery, hormone therapy use, parity, age at menarche, breastfeeding history, age at first birth, oral contraceptive use, smoking and age. All analyses will be stratified according to menopausal status.

Due the repeated nature of the data a multilevel regression model will be performed to examine the relationship between changes over time in outcome measures, such as body composition, physical activity and dietary intake, and the metabolic syndrome while controlling for other confounding variables. Significance at p<0.05 will be assumed and SPSS will be used for statistical analysis.

### Data collection

Participant recruitment will be co-ordinated by EG and SMcG. Data will be collected by EG and processed by EG, SMcG and LH. Standardized testing and data processing protocols have been developed to ensure long-term valid data collection methods. Long-term management of the data collection and processing will be directed by L.C. and JH (principle investigators). All measurements and data processing will be completed at the Trinity Centre for Health Sciences, St. James’s Hospital, Dublin, Ireland.

## Discussion

The results of this prospective cohort study will provide valuable information regarding the risk reducing potential of modifiable risk factors for breast cancer in unaffected *BRCA1* and *BRCA2* gene carriers. To date, no study has prospectively examined lifestyle risk factors in this group, nor have the biological mechanisms linking obesity and physical inactivity to breast cancer risk been investigated. This study will provide information regarding whether modifiable factors including body composition, the metabolic syndrome, physical activity and dietary intake can modulate breast cancer risk in *BRCA* mutation carriers, whether risk of breast cancer occurrence can be predicted in these individuals by PBMC telomere length and whether telomere length in these individuals is associated with various physical activity and lifestyle factors.

## Abbreviations

BMI: Body mass index; PCR: Polymerase chain reaction; TC: Total cholesterol; HDL-C: High-density lipoprotein cholesterol; LDL-C: Low-density lipoprotein cholesterol; TG: Triglycerides; HBA1c: Glycosylated haemoglobin; CRP: C – reactive protein; cm: Centimetre; mmol.L-1: Millimoles per litre; mmHG: Millimetres mercury; mg.dL-1: Milligrams per decilitre; MLTPAQ: Minnesota leisure time physical activity questionnaire; TOPAQ: Tecumseh occupational physical activity questionnaire; MET-min/day: Metabolic equivalent minutes per day; Kg.m-2: Kilograms per metre squared; FFQ: Food frequency questionnaire; BMR: Basal metabolic rate.

## Competing interests

The authors declare that they have no competing interests.

## Authors’ contributions

EG, JH, JOS and EC developed the idea for this study. EG was responsible for drafting the manuscript with contributions from SMcG and LH. EG is responsible for measurement and analysis of body composition, physical activity and metabolic syndrome outcomes. SMcG is responsible for DNA processing and analysis and for measurement of DNA telomere length. LH is responsible for assessment and analysis of dietary outcomes. KB provided statistical advice and contributed to the study design. All authors approved the final version of the manuscript.

## Pre-publication history

The pre-publication history for this paper can be accessed here:

http://www.biomedcentral.com/1471-2407/13/138/prepub
